# Environmental Toxic Elements and Atherosclerotic Disease: The Epigenetic Connection

**DOI:** 10.3390/life16101751

**Published:** 2026-10-19

**Authors:** Ludovica Simonini, Francesca Gorini, Mariangela Palazzo, Fabrizio Minichilli, Andrea Borghini

**Affiliations:** Institute of Clinical Physiology, National Research Council, 5624 Pisa, Italy; ludovicasimonini@cnr.it (L.S.); mariangelapalazzo@cnr.it (M.P.); fabrizio.minichilli@cnr.it (F.M.)

**Keywords:** toxic elements, environmental exposure, atherosclerosis, atherosclerotic cardiovascular disease, epigenetics, DNA methylation, non-coding RNAs, microRNAs

## Abstract

Toxic elements (TEs) are widespread environmental contaminants that act as systemic toxicants and are recognized contributors to atherosclerotic cardiovascular disease (ASCVD) morbidity and mortality, posing significant health risks even at low levels of exposure. Increasing evidence indicates that, beyond their direct genotoxic effects, TEs exert pathogenic actions through epigenetic mechanisms that regulate gene expression without altering the underlying DNA sequence. Major epigenetic modifications associated with TE exposure include aberrant DNA methylation and dysregulation of non-coding RNAs, particularly microRNAs (miRNAs). These modifications may disrupt key vascular cellular processes involved in atherogenesis, including endothelial dysfunction, oxidative stress responses, inflammatory signaling, and lipid metabolism, thereby promoting the initiation and progression of atherosclerotic lesions. The aim of this literature review is to critically examine the evidence linking TE exposure to atherosclerosis, with a particular focus on the potential role of epigenetic mechanisms in mediating this association. Specifically, it summarizes the epidemiological evidence linking TE exposure to ASCVD, discusses the mechanisms through which TE-induced epigenetic alterations may contribute to the development and progression of atherosclerotic disease, and critically evaluates the strengths and limitations of the available evidence to identify priorities for future research.

## 1. Introduction

Atherosclerotic cardiovascular disease (ASCVD) remains the leading cause of mortality worldwide and is increasingly recognized as a multifactorial disorder in which environmental exposures play a central role in modulating disease susceptibility [1]. In addition to traditional cardiovascular risk factors, a growing body of evidence highlights the contribution of toxic environmental metals, including arsenic (As), cadmium (Cd), lead (Pb), and mercury (Hg), as relevant non-classical determinants of cardiovascular pathology [2]. These elements are ubiquitous environmental contaminants, and human exposure occurs through contaminated water, air pollution, dietary intake, and occupational settings [3].

Epidemiological studies have consistently demonstrated associations between exposure to toxic elements (TEs) and increased cardiovascular morbidity and mortality. In particular, exposure to several TEs has been linked to ASCVD development [4]. Meta-analyses have reported elevated cardiovascular risk following Pb, Cd, and As exposure, while more recent pooled evidence has also confirmed a relationship between mercury exposure and cardiovascular disease (CVD) incidence [5]. In contrast, evidence regarding hexavalent chromium remains more limited and inconsistent, highlighting the need for further well-designed epidemiological studies [6].

Mechanistically, TEs exert their deleterious cardiovascular effects through direct oxidative and inflammatory injury, as well as through more complex molecular pathways involving epigenetic regulation [7,8,9]. Epigenetic mechanisms refer to heritable and reversible modifications of gene expression that occur without alterations in the DNA sequence and include DNA methylation, histone post-translational modifications, and the regulation of non-coding RNAs, including microRNAs (miRNAs), circular RNAs (circRNAs), and long non-coding RNAs (lncRNAs) [10,11]. These mechanisms are increasingly recognized as potential mediators of environmental exposures on cardiovascular health [12]. In the context of TE exposure, multiple studies have demonstrated consistent alterations in gene-specific DNA methylation patterns, together with dysregulation of chromatin structure and miRNA expression profiles, supporting the role of these epigenetic modifications as key mechanistic links between environmental exposure and atherosclerotic disease [13,14,15,16,17].

In an effort to bring clarity to this complex yet highly relevant topic, we critically synthesize current evidence on the epigenetic effects of TEs in the context of ASCVD. Specifically, this literature review summarizes the epidemiological evidence linking exposure to these TEs to ASCVD and related cardiovascular outcomes, examines the evidence supporting the involvement of epigenetic alterations, particularly DNA methylation and non-coding RNA dysregulation, in this association, and evaluates the extent to which findings from human epidemiological studies and experimental models support the biological plausibility of these mechanisms. Furthermore, this review discusses the main limitations and strengths in the current literature and identifies priorities for future research, including the validation of epigenetic signatures as potential biomarkers of exposure and early cardiovascular alterations.

## 2. Literature Search Strategy

A bibliographic search was conducted in July 2026 to identify studies investigating the relationship between exposure to TEs, epigenetic alterations, and ASCVD. The search was performed using PubMed, with combinations of terms related to TEs, terms related to epigenetic mechanisms, and terms related to atherosclerosis and cardiovascular disease. PubMed was selected because it provides comprehensive coverage of biomedical and epidemiological literature relevant to the scope of this literature review. The complete search query used in PubMed is reported below to facilitate reproducibility: (“Arsenic”[Mesh] OR arsenic[tiab] OR “Cadmium”[Mesh] OR cadmium[tiab] OR “Lead”[Mesh] OR lead[tiab] OR “Mercury”[Mesh] OR mercury[tiab] OR “Toxic Metals”[Mesh] OR “Heavy Metals”[tiab] OR “toxic element*”[tiab]) AND (epigenetic*[tiab] OR “DNA Methylation”[Mesh] OR methylation[tiab] OR “Histone Code”[Mesh] OR histone*[tiab] OR “histone modification*”[tiab] OR “histone modifications”[tiab] OR “MicroRNAs”[Mesh] OR microRNA*[tiab] OR miRNA*[tiab] OR “non-coding RNA*”[tiab] OR ncRNA*[tiab]) AND (“Atherosclerosis”[Mesh] OR atheroscleros*[tiab] OR “atherosclerotic cardiovascular disease*”[tiab] OR ASCVD[tiab] OR “Coronary Artery Disease”[Mesh] OR CAD[tiab] OR “coronary heart disease”[tiab])”.

To ensure broad coverage of the available evidence, no restrictions were applied regarding publication type or study design. Articles published in English and relevant to the scope of the review were considered, including original research studies, experimental studies, reviews, and meta-analyses. Studies were selected based on their relevance to the relationship between TE exposure, epigenetic mechanisms, and cardiovascular or atherosclerotic outcomes. The reference lists of selected articles and relevant reviews were also screened to identify additional relevant studies not retrieved through the primary database search.

## 3. Toxic Elements and Cardiovascular Risk

Humans are routinely exposed to a broad spectrum of trace elements, including heavy metals and metalloids, owing to their ubiquitous presence in the environment and their uptake through food, drinking water, inhalation, as well as, to a lesser extent, through dermal contact and occupational exposure [18]. Although trace elements are naturally occurring components of the Earth’s crust and are primarily released through natural processes such as rock weathering and soil erosion, their widespread environmental contamination has emerged as one of the major environmental challenges of the twenty-first century [18,19,20]. Indeed, rapid industrialization and expanding anthropogenic activities have markedly increased the release of heavy metals into the environment through industrial emissions, agricultural practices, fossil fuel combustion, waste disposal, and other human-related sources [21].

Owing to their resistance to chemical and biological degradation, these contaminants persist in the environment, undergo transformations into various toxic forms, bioaccumulate in living organisms, and biomagnify through the food chain [18,19]. Consequently, beyond posing a significant threat to environmental ecosystems, they constitute a major public health concern [20,21]. Of note, the impact of metals and metalloids on the environment and human health depends either on their concentration, as observed for essential or beneficial trace elements such as nickel, selenium, zinc, cobalt, and copper, which may become toxic when present above physiological thresholds, or on their intrinsic toxicity, as in the case of non-essential elements including As, Cd, Pb, and Hg, which are ranked among the priority hazardous substances identified by the Agency for Toxic Substances and Disease Registry [20,22]. In parallel, the World Health Organization (WHO) has classified As, Cd, Pb, and Hg as chemicals of major public health significance [23].

In recent years, human biomonitoring (HBM) has been increasingly acknowledged as a valuable tool for assessing human exposure to trace elements and investigating their potential effects on human health, regardless of the specific exposure sources or pathways involved [18,24]. Blood and urine are the most widely accepted matrices for biomonitoring exposure to trace elements [24]. However, urine is often preferred in large-scale HBM studies because it can be collected non-invasively and with relative ease, particularly in vulnerable populations such as children [25]. Blood levels, for which well-established reference ranges and occupational exposure limits are available, are commonly used to assess occupational exposure to Pb and elemental and inorganic Hg and are particularly useful for evaluating relatively recent exposure [26]. Urinary Cd concentrations reflect long-term exposure and renal accumulation due to the long half-life of this metal, whereas urinary As speciation represents a particularly suitable indicator of recent exposure to inorganic arsenic and of methylation capacity [26]. Hair and nails serve as matrices for evaluating longer-term or historical exposures, although the results should be interpreted with caution because of interindividual differences in hair growth [26]. Nonetheless, methylmercury concentrations in hair generally reflect exposure over periods ranging from weeks to months, whereas As levels in hair or nails can indicate past exposure over periods of up to several years and are useful in forensic and epidemiological studies [26].

Over the last two decades, accumulating evidence has highlighted the role of environmental exposure to TEs in increasing the risk of ASCVD.

Exposure to As, which is formally classified as a metalloid, may occur during the processing of glass, textiles, ammunition, and paper, as well as through the production or use of wood preservatives and cosmetics [19]. Inorganic As species are generally more toxic than methylated forms and are classified as Group 1 human carcinogens [27,28]. Contaminated water used for drinking, food preparation, and irrigation of food crops represents the greatest public health threat associated with As exposure [29], as more than 230 million people worldwide are exposed to excessive levels of As in drinking water, particularly in regions such as East and Southeast Asia and the Indian subcontinent [30,31]. High chronic arsenic exposure (>50 μg/L in drinking water) has been associated with CVD, including ischemic heart disease (IHD), and peripheral arterial disease (PAD), with corresponding excess risks of 32%, 89%, and 217%, respectively [32]. In contrast, the evidence regarding the cardiovascular effects of low-to-moderate As exposure remains inconclusive [32]. More recently, low-to-moderate urinary inorganic As levels have been associated with a modest increase in carotid intima–media thickness, a widely used surrogate marker of ASCVD, as well as with the extent of carotid atherosclerosis [33,34]. However, no statistically significant association was observed between urinary As levels and the presence of carotid plaques after adjustment for other cardiovascular risk factors [33].

Cd, which occurs naturally in soil, minerals, and water, is a highly reactive metal used in nickel–cadmium batteries, metal alloys, pigments, plastic stabilizers, dyes, paints, glass production, and electroplating [35,36]. Beyond occupational activities, active and passive smoking represent additional non-occupational sources of Cd exposure, whereas the dietary intake of contaminated food constitutes the main exposure pathway among non-smokers [37,38]. In addition to its well-established association with Itai-Itai disease, a severe form of Cd toxicity first identified in Japan and characterized by renal dysfunction and osteomalacia, Cd has been classified as a Group 1 carcinogen by the International Agency for Research on Cancer [28,36]. Furthermore, owing to its long biological half-life in humans (up to 25–30 years), Cd can accumulate in the body over time and is associated with multiple toxic effects, including CVD [35,39,40]. A meta-analysis of cross-sectional studies reported that higher blood Cd levels have been associated with an increased prevalence of IHD (odds ratio (OR) = 1.59, 95% confidence interval (CI): 1.24–2.04); however, this relationship lost statistical significance when restricted to only non-smokers [41]. Similarly, a meta-analysis of prospective studies reported an association between higher Cd exposure and increased IHD mortality (hazard ratio (HR) = 1.60, 95%CI: 1.21–2.10) [41]. More recently, a meta-analysis found a monotonic positive association between urinary Cd exposure and the risk of IHD, with a pooled relative risk (RR) of 1.82 (95%CI: 1.16–2.86), supporting the detrimental impact of this metal on cardiovascular health [40].

Although Pb occurs naturally in trace amounts in the Earth’s crust, it has become a widespread and persistent environmental contaminant largely as a result of anthropogenic activities [42,43]. Over the past several decades, the global ban on leaded gasoline has led to a more than 90% reduction in mean blood lead concentrations in the US general population [43,44]. Nevertheless, Pb remains a major public health concern because of its toxicity, high bioaccumulation potential, and classification as a Group 2A human carcinogen [43]. Its persistence in the environment, resulting from historical vehicle emissions, together with continued contamination from sources such as mining, battery manufacturing, smelting, electronic waste recycling, and canned food production, contributes to ongoing human exposure [42,45]. Dietary intake is considered the primary route of Pb exposure, whereas inhalation of lead-containing dust represents an important exposure pathway for both workers and the general population [43]. Growing evidence supports the role of Pb as a risk factor for hypertension, cardiovascular outcomes (including IHD and PAD), and CVD mortality at non-occupational, community-levels of exposure [46,47]. A meta-analysis demonstrated a linear relationship between blood Pb concentrations and IHD risk, with an estimated RR of 1.07 (95%CI: 1.04–1.10) for every 5 μg/dL increase in blood Pb levels [48]. Furthermore, in a population-based cohort study, individuals in the highest quartile of blood Pb levels had an 8% higher risk of plaque compared with those in the lowest quartile [49]. The extent of atherosclerotic plaque also increased with increasing blood Pb concentrations, supporting a potential role in ASCVD [49].

Hg, which is naturally released into the environment by forest fires and volcanic activity, is also emitted from anthropogenic sources such as iron and steel production, cement manufacturing, coal-fired power plants, and waste incineration, which collectively account for approximately two-thirds of global Hg emissions [50,51]. Hg occurs in three primary forms: elemental mercury, which is liquid at room temperature; inorganic mercury, which is mainly present in the divalent state; and organic mercury, particularly methylmercury, a globally distributed and highly toxic contaminant that poses risks to both humans and wildlife due to its ability to bioaccumulate and biomagnify through food webs [50,51,52]. The Minamata Convention, aimed at controlling, reducing, and eliminating anthropogenic releases of Hg throughout its life cycle, was adopted in 2013 and entered into force in 2017 [53]. In 2025, it established a global phase-out of dental amalgam, which can release small amounts of mercury vapor, by 2034 [54,55]. A number of epidemiological studies suggest that Hg can negatively affect cardiovascular function [21]. According to a recent meta-analysis, Hg exposure was associated with an increase in nonfatal IHD (RR = 1.21, 95%CI: 0.98–1.50), CVD mortality (RR = 1.68, 95%CI: 1.15–2.45), and mortality due to other heart diseases (RR = 1.50, 95%CI: 1.07, 2.11) [56], although the associations were of borderline statistical significance. However, interpretation of these findings should consider that most studies assessed total Hg concentrations, limiting the ability to distinguish the cardiovascular effects of specific Hg species [56].

**Figure 1:**
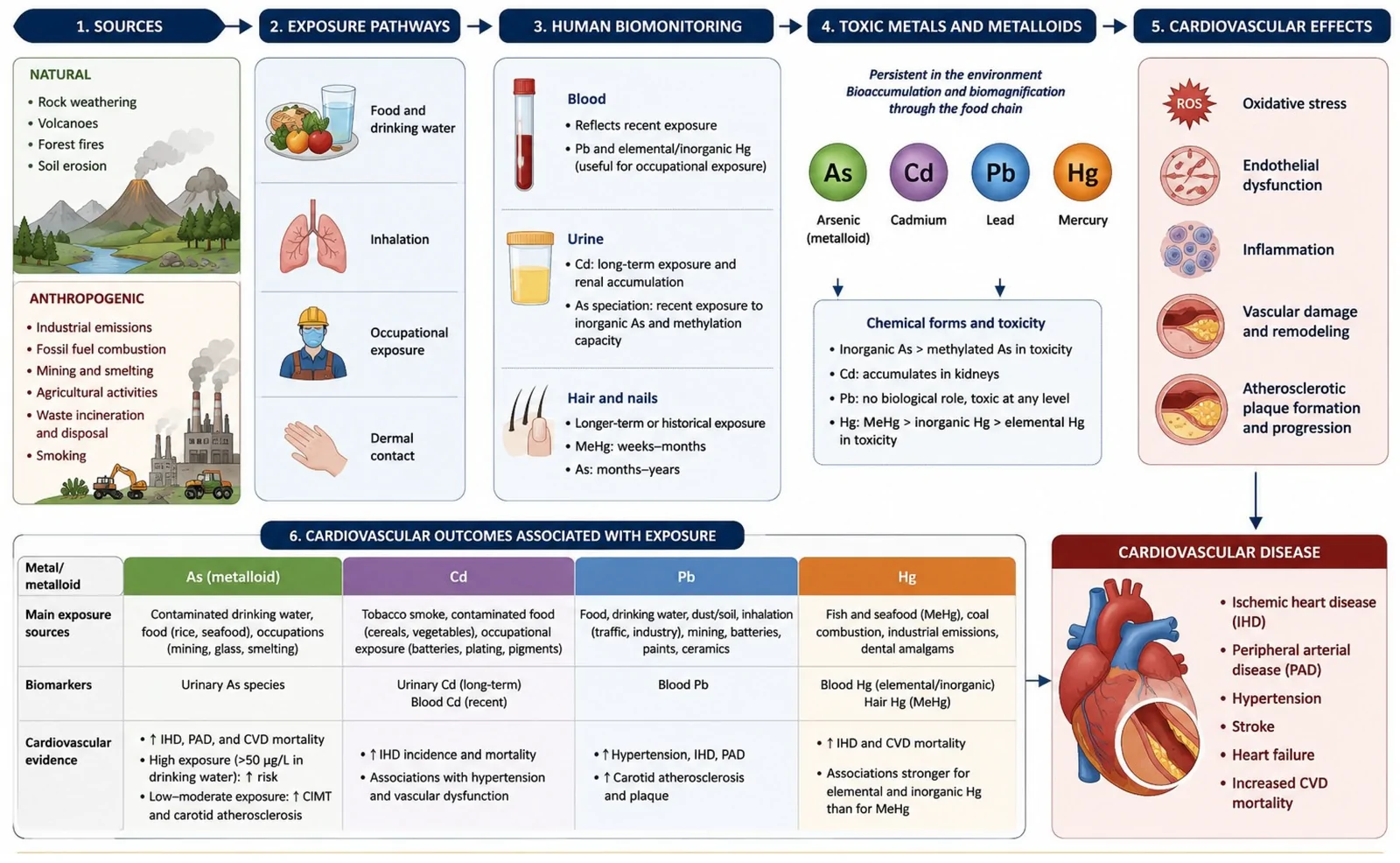
Environmental exposure to toxic elements and cardiovascular health. Natural and anthropogenic sources contribute to human exposure to arsenic (As), cadmium (Cd), lead (Pb), and mercury (Hg) through multiple pathways. Blood, urine, hair, and nails can be used for human biomonitoring, providing information on recent, cumulative, or historical exposure. These persistent contaminants may bioaccumulate and biomagnify and contribute to oxidative stress, endothelial dysfunction, inflammation, vascular damage, and atherosclerotic plaque formation, potentially increasing the risk of cardiovascular diseases. The cardiovascular impact depends on exposure level, chemical species, biomarker/matrix, outcome considered, individual susceptibility, and co-exposures. Figure was generated using ChatGPT (version 5.5).

Collectively, the available evidence indicates that As, Cd, Pb, and Hg may contribute to CVD, including IHD, although the strength and consistency of these associations vary according to the exposure levels, chemical species, biomarkers and matrices used for exposure assessment, and the specific cardiovascular outcome considered (Figure 1).

## 4. Toxic Elements and Epigenetic Mechanisms in Atherosclerosis

Increasing evidence indicates that environmental exposure to TEs may contribute to CVD, including atherosclerosis, through mechanisms involving oxidative stress, endothelial dysfunction, inflammation, and epigenetic alterations [57]. Epigenetic regulation encompasses a complex network of processes, including DNA methylation, non-coding RNAs, histone post-translational modifications, and changes in chromatin organization and accessibility. These mechanisms are particularly relevant to atherosclerosis because they regulate genes involved in inflammation, oxidative stress, lipid homeostasis, endothelial function, vascular smooth muscle cell phenotype, and immune responses. TEs may interfere with these regulatory pathways through interconnected mechanisms, including oxidative stress, altered activity of epigenetic enzymes, disruption of cellular signalling, and changes in chromatin structure, potentially leading to persistent alterations in vascular cell function [15,58].

Recent evidence supports a gene–environment framework in which environmental pollutants may interact with inherited susceptibility through epigenetic mechanisms, including alterations in DNA methylation, chromatin organization, and non-coding RNA activity [59]. These changes may constitute a molecular interface between environmental exposure and genetic predisposition, contributing to persistent dysregulation of pathways involved in inflammation, lipid metabolism, oxidative stress, and vascular homeostasis. Transgenerational epigenetic effects have also been reported, although their relevance to human CVD remains uncertain [59]. Accordingly, epigenetic dysregulation is increasingly recognized as a crucial component of environmentally induced cardiovascular toxicity. Elucidating how exposure to TEs influences these mechanisms may provide further insight into the molecular pathways linking environmental exposure to vascular dysfunction and atherosclerotic disease [21]. An important consideration when interpreting TE-associated epigenetic alterations is their dependence on the tissue and biological material analyzed. Epigenetic profiles are highly cell- and tissue-specific, reflecting differences in cellular composition, gene expression, and functional state. Consequently, alterations detected in peripheral blood may not fully reflect those occurring in vascular endothelial cells, vascular smooth muscle cells, macrophages within atherosclerotic plaques, or other tissues involved in cardiovascular disease. Furthermore, the biological information obtained from whole blood, plasma or serum, urine, and tissue samples differs according to the cellular or molecular components analyzed. These differences should be considered when comparing findings across studies and assessing the potential relevance of epigenetic signatures to vascular pathology.

### 4.1. DNA Methylation

DNA methylation is the most extensively investigated epigenetic mechanism linking TE exposure to ASCVD. Current evidence indicates that exposure to TEs can induce both global and locus-specific alterations in DNA methylation, potentially affecting genes and pathways involved in lipid metabolism, inflammation, oxidative stress, and vascular homeostasis. Importantly, these alterations are not uniform: TE exposure may induce global DNA hypomethylation while simultaneously promoting hypermethylation at specific genomic loci, indicating that their biological consequences depend on the metal involved, exposure characteristics, tissue or cell type, and genomic context [16,60,61,62,63,64].

Human epidemiological studies provide evidence linking metal exposure and altered DNA methylation to early vascular changes. In the Strong Heart Study, Domingo-Relloso et al. [60] investigated whether As exposure was associated with ASCVD and whether DNA methylation could mediate this association. The study included 2321 American Indian participants (mean age, 56.2 years; 58.6% women). DNA methylation was assessed in peripheral blood, while urinary As species were quantified by high-performance liquid chromatography coupled with inductively coupled plasma mass spectrometry.

The authors identified 20 differentially methylated positions (DMPs) that potentially mediated the association between arsenic exposure and incident ASCVD, and 13 DMPs that potentially mediated the association with mortality. Several of these DMPs were annotated to genes involved in glucose metabolism, suggesting that metabolic dysfunction may play a role in arsenic-related cardiovascular toxicity. Notably, 11 of the identified DMPs showed similar associations with ASCVD in three independent prospective cohorts: the Framingham Heart Study, the Women’s Health Initiative, and the Multi-Ethnic Study of Atherosclerosis.

The epidemiological findings were further supported by experimental evidence from a mouse model. In ApoE−/− mice exposed to As during early life, hepatic differentially methylated regions (DMRs) significantly overlapped with arsenic-associated methylation changes identified in humans. Concordant DMRs involved 20 genes, while concordant DMPs involved 10 genes. Taken together, these findings suggest that differential DNA methylation may represent a biological pathway linking As exposure to CVD. They indicate that metabolic pathways, particularly those involved in glucose homeostasis and diabetes, may contribute to arsenic-associated cardiovascular toxicity [60].

Further support comes from a recent single-cell multi-omic study in ApoE−/− mice. Makhani et al. [61] showed that chronic As exposure induced extensive changes in chromatin accessibility in plaque macrophages, with several differentially accessible regions overlapping genes previously identified as differentially methylated in arsenic-exposed participants from the Strong Heart Study, including Col1a1, Tgfbr1, and Nav2. Although DNA methylation was not directly assessed, the convergence of methylation and chromatin accessibility changes at these loci provides complementary evidence that arsenic-associated epigenetic alterations may influence the regulation of genes involved in macrophage function and atherosclerotic plaque biology [61].

The potential role of DNA methylation as a mechanistic link between metal exposure and vascular injury is also supported by evidence from studies of combined metal exposure. Lin et al. [16] investigated 738 participants aged 12–30 years, measuring urinary Pb and Cd concentrations, global DNA methylation using the 5-methyl-2′-deoxycytidine/deoxyguanosine ratio, and carotid intima-media thickness (CIMT) as markers of subclinical atherosclerosis. Pb exposure was associated with altered global DNA methylation and increased CIMT, whereas Cd exposure was associated with increased CIMT alone. Notably, the association between Pb exposure and CIMT was more pronounced among participants with higher global DNA methylation, suggesting that methylation status may modify the relationship between Pb exposure and early vascular remodeling [16].

Using an in silico approach, Riffo-Campos et al. [62] investigated the relationship between exposure to TEs (As, Cd, antimony, and tungsten), epigenetic alterations, and CIMT in middle-aged men from the Aragon Workers’ Health Study. Urinary Cd and tungsten concentrations were positively associated with femoral and carotid IMT. Importantly, 46% of the genes located near metal-associated DMRs also overlapped with genes associated with atherosclerosis-related DMRs, with functional enrichment pointing to inflammatory, metabolic, and transport-related pathways. Protein–protein interaction analysis further identified shared network hubs linking metal-associated and atherosclerosis-associated proteins. Despite the small sample size and exploratory design, this intriguing study suggests that TE exposure and subclinical atherosclerosis may converge on partially overlapping epigenetic networks [62].

Mechanistic evidence from cell-based models provides further insight into how metal-induced methylation changes may directly affect atherogenic pathways. Song et al. [63] investigated the effects of As exposure on DNA methylation and cholesterol efflux in THP-1 macrophages. Arsenic exposure increased reactive oxygen species (ROS) generation and induced both the transcription and activity of DNA methyltransferase 1 (DNMT1). This was accompanied by hypermethylation of the promoter region of ATP-binding cassette transporter A1 (ABCA1), a key regulator of macrophage cholesterol efflux, resulting in reduced ABCA1 expression and impaired cholesterol efflux. Interestingly, As simultaneously depleted the methyl donor S-adenosylmethionine and induced global DNA hypomethylation. Thus, As produced apparently opposing epigenetic effects, characterized by global hypomethylation together with locus-specific hypermethylation of ABCA1. Pharmacological experiments further supported this mechanism. Reducing oxidative stress with N-acetylcysteine or inhibiting DNMT1 activity with 5-aza-2′-deoxycytidine attenuated the As-induced impairment of cholesterol efflux, whereas supplementation with S-adenosylmethionine (SAM) had no protective effect. These findings suggest that ROS generation acts upstream of DNMT1 activation and ABCA1 promoter hypermethylation, providing a mechanistic link between As exposure, epigenetic dysregulation, impaired cholesterol efflux, and a potential increase in macrophage foam-cell formation [63].

Biochemical evidence further supports the ability of TEs to interfere directly with the enzymatic machinery controlling DNA methylation. Poirier and Vlasova investigated the effects of Cd and Zn on DNMT1 activity in hepatic nuclear extracts from rats maintained on methyl-deficient or methyl-supplemented diets. Both elements inhibited DNMT1 activity, but Cd consistently produced a stronger inhibitory effect than Zn. Moreover, the kinetics of Cd-mediated inhibition differed according to dietary methyl-group availability, suggesting that cellular methyl metabolism may modulate the susceptibility of DNMT1 to metal toxicity. These findings provide a mechanistic basis for the hypothesis that Cd can interfere with DNA methylation through direct effects on methyltransferase activity, in addition to perturbing cellular methyl-group metabolism [64].

While most available evidence focuses on metal exposure during adulthood, studies of developmental exposure suggest that the timing of exposure may influence the extent and the specific genomic regions affected by epigenetic alterations.

Ngalame et al. [65] investigated prenatal As exposure in ApoE−/− mice. Pregnant mice received arsenic in drinking water from gestational day 8 to 18, and DNA methylation was evaluated in fetuses and offspring at 3, 10, and 24 weeks of age. Although global DNA methylation increased with age in both exposed and control animals and was not affected by arsenic exposure, locus-specific analysis in 10-week-old offspring revealed increased methylation within the transcribed region of Hsp70 [65]. Similarly, Svoboda et al. [66] reported widespread, locus-specific DNA methylation changes in the hearts of adult offspring following perinatal Pb exposure, with marked sex-specific patterns and enrichment of genes involved in cardiac and tissue development. Together, these findings suggest that global methylation measures may not fully capture locus-specific epigenetic alterations induced by developmental metal exposure. Moreover, whether such alterations persist over time and contribute directly to atherosclerosis remains to be established. The timing of exposure may be particularly relevant because both prenatal and perinatal development represent critical windows of susceptibility during which epigenetic mechanisms contributes to the constitution of tissue-specific gene expression patterns and long-term physiological functions. Exposure to TEs during these phases may interfere with developmental programming and induce epigenetic modifications that persist beyond the initial exposure, potentially influencing metabolic and cardiovascular homeostasis later in life. In this context, the findings reported by Ngalame et al. and Svoboda et al. suggest that developmental exposure to As and Pb can affect DNA methylation patterns in offspring, although the direct contribution of these changes to adult-onset ASCVD has not been established [65,66].

Collectively, the available evidence supports a role for DNA methylation as a potential epigenetic link between TE exposure and vascular injury. Across human, animal, and cell-based studies, TE exposure has been associated with alterations in DNA methylation that may affect pathways involved in lipid metabolism, inflammation, oxidative stress, and vascular homeostasis. These effects appear to be context-dependent, varying according to the specific element, exposure timing, tissue or cell type, and genomic locus. However, further studies are needed to establish the persistence of these epigenetic alterations and determine whether they causally contribute to the development and progression of ASCVD.

### 4.2. Non-Coding RNAs (ncRNAs)

Non-coding RNAs (ncRNAs) comprise several classes of regulatory RNA molecules, including microRNAs (miRNAs), long non-coding RNAs (lncRNAs), and circular RNAs (circRNAs). These molecules are increasingly recognized as important regulators of cardiovascular homeostasis and disease, acting through diverse mechanisms that include post-transcriptional regulation of gene expression and modulation of epigenetic processes. miRNAs have increasingly emerged as potential mediators of vascular effects of TE exposure. Rather than acting through a single molecular pathway, metal-induced miRNA dysregulation appears to affect several processes central to atherosclerosis. Evidence from experimental models and human biomonitoring studies has shown that exposure to Cd, As, Hg and Pb can alter the expression of specific miRNAs involved in cholesterol metabolism, endothelial function, inflammation, and angiogenesis [14,67,68,69,70,71].

One of the most direct links between TE exposure, miRNA dysregulation, and atherosclerosis has been demonstrated for Cd [14]. In ApoE−/− mice fed a high-fat diet, Cd exposure accelerated atherosclerotic progression and was accompanied by marked alterations in systemic and vascular lipid metabolism. Integrated transcriptomic, metabolomic, and miRNA analyses identified miRNA-30d-5p and miRNA-504-3p as candidate regulators of this response. Cd increased miRNA-30d-5p, which directly targets neutral cholesteryl ester hydrolase 1, while reducing miRNA-504-3p, which targets the scavenger receptor CD36. The resulting imbalance favours cholesterol uptake and impairs intracellular cholesterol hydrolysis, thereby promoting lipid accumulation in macrophages and foam-cell formation. This mechanistic relationship was also supported by human data. In a case–control study of 494 patients with ischemic stroke and 494 matched controls, circulating miRNA-30d-5p levels were positively associated with Cd exposure and partially mediated the Cd-stroke association. Together with experimental evidence linking metal exposure to altered cholesterol handling and foam-cell formation, these findings support a potential link between metal-induced miRNA dysregulation, impaired lipid homeostasis, and ASCVD [14].

Similar miRNA-mediated effects have also been reported for As, which has been shown to interfere with endothelial angiogenic responses. In ApoE−/− mice exposed to As in utero, States et al. [67] identified persistent changes in hepatic gene-expression networks in the offspring, together with altered expression of putative miRNAs targeting HSP70-related transcripts. Although these findings do not establish a direct miRNA-mediated mechanism of accelerated plaque formation, they support the concept that prenatal As exposure can reprogram regulatory networks involved in inflammation and metabolism, potentially contributing to the later-life susceptibility to atherosclerosis observed in this model [67].

Experimental studies provide further insight into the candidate mechanisms underlying miRNA-mediated vascular effects of As exposure. Gao et al. [68] examined the anti-angiogenic effects of As exposure, which may be relevant to the microvascular dysfunction observed in atherosclerosis. In human umbilical vein endothelial cells, As inhibited proliferation, migration, and capillary tube formation, and reduced miRNA-425-5p expression both in vitro and in vivo. Overexpression of miRNA-425-5p reversed the anti-angiogenic effects of As by targeting CCM3, which was upregulated following As exposure. Alterations in Notch and VEGF/p38 signalling were also involved, suggesting a miRNA-425-5p/CCM3/Notch–VEGF axis underlying the vascular effects of As [68].

Moreover, evidence for a potential role of miRNA dysregulation in the vascular effects of Pb exposure comes from a more recent study [69] investigating circulating miRNA-126 and miRNA-155 in a Mexican population environmentally exposed to Pb. The authors examined their predicted target genes to identify potentially affected biological pathways. Both miRNAs were significantly associated with higher blood Pb concentrations, while target-gene enrichment analysis highlighted pathways involved in inflammation, angiogenesis, lipid metabolism, and atherosclerosis, including the AGE–RAGE, TNF, Toll-like receptor, and fluid shear stress signalling pathways [69].

Alterations in miRNA profiles have also been reported after exposure to TE mixtures, suggesting that metal-induced epigenetic dysregulation may involve complex interactions among multiple miRNAs and their target pathways [70,71]. Kong et al. [70] examined the relationship between circulating miRNAs and urinary TEs (As, Hg, Cd, and Pb) in 60 Hong Kong adolescents aged 12–19 years with microalbuminuria, compared with 60 age- and sex-matched normoalbuminuric controls. Of the four miRNAs tested, only miRNA-21 was significantly associated with microalbuminuria. Urinary As and Pb were both negatively associated with miRNA-21 and miRNA-221. Although no direct association was observed between metal levels and microalbuminuria, these findings suggest that metal exposure may influence miRNA profiles associated with early vascular and renal dysfunction [70]. Furthermore, bioinformatic approaches have been used to explore molecular networks potentially affected by combined metal exposure. Nguyen and Kim modeled the effects of a Cd–Pb–Hg mixture and identified genes involved in inflammation, apoptosis, and lipid metabolism, along with six metal-associated miRNAs. The predicted regulatory networks were enriched in pathways relevant to atherosclerosis, including oxidative stress, lipoprotein metabolism, fluid shear stress, and IL-6 signalling [71].

Collectively, the evidence indicates that TE exposure can perturb miRNA-mediated regulatory networks involved in key processes underlying vascular disease, including lipid handling, inflammation, endothelial function, and angiogenesis. These effects have been observed across experimental models and human studies, with some evidence supporting links between specific miRNAs, metal exposure, and vascular phenotypes. Table 1 summarizes the studies investigating DNA methylation and non-coding RNA alterations in relation to TE exposure and their potential impact on ASCVD. 

## 5. Conclusions

### 5.1. Summary of the Main Findings

Epigenetic dysregulation may represent an important molecular connection between TE exposure and ASCVD, linking environmental exposure to alterations in pathways involved in vascular homeostasis and atherogenesis.

Among the epigenetic mechanisms investigated to date, DNA methylation and miRNA dysregulation are the best characterized. Evidence from human, animal, and cell-based studies indicates that TE-associated epigenetic alterations may affect multiple processes relevant to atherosclerosis, including inflammation, oxidative stress, endothelial function, angiogenesis, lipid metabolism, and macrophage cholesterol handling. Notably, the reported effects of DNA methylation appear to depend on the genomic locus, tissue or cell type, and exposure characteristics, while miRNA dysregulation may influence interconnected pathways involved in lipid homeostasis and vascular function. Although these findings support a potential role for epigenetic mechanisms in linking TE exposure to vascular injury, the extent to which these alterations directly contribute to ASCVD development and progression remains to be established.

However, evidence remains uneven across epigenetic mechanisms, with other classes of non-coding RNAs receiving considerably less attention. In particular, circRNAs and lncRNAs remain largely unexplored in the context of TE exposure and ASCVD, despite their potential roles in regulating gene expression and pathways relevant to vascular function and atherosclerosis. Investigating these RNA classes, together with their interactions with miRNAs and other epigenetic mechanisms, may help identify additional regulatory layers through which TE exposure influences cardiovascular health.

**Figure 2:**
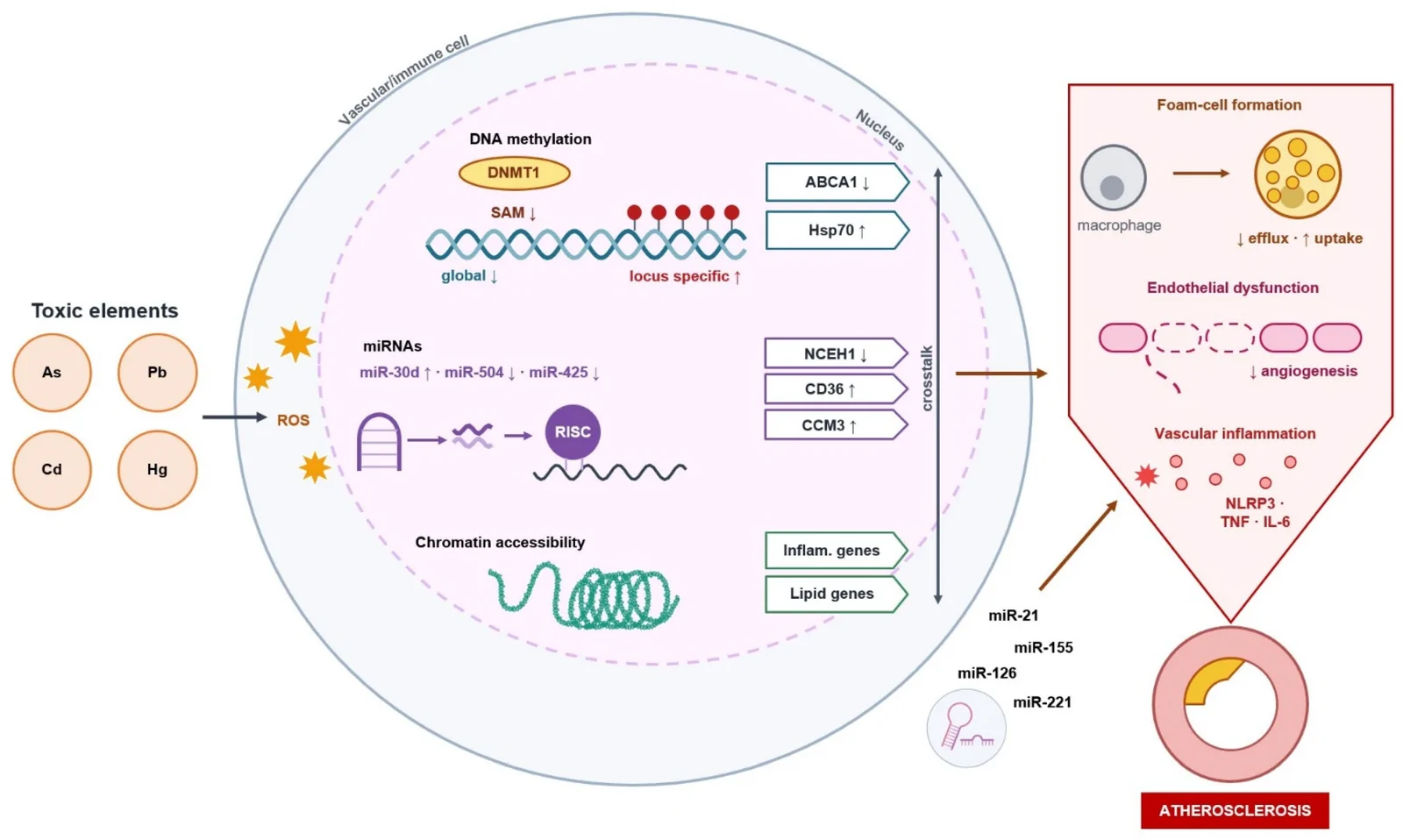
Conceptual representation of the potential mechanisms linking toxic element (TE) exposure to atherosclerotic disease, with a focus on epigenetic dysregulation, including DNA methylation and miRNA-expression alterations, and its potential involvement in pathways contributing to the development and progression of atherosclerosis.

Importantly, the cardiovascular effects of TE exposure should be considered within the broader context of both traditional cardiovascular risk factors and other environmental and individual determinants of disease susceptibility. Smoking, hypertension, diabetes, and dyslipidemia may interact with TE-induced epigenetic alterations affecting oxidative stress, inflammation, endothelial function, and lipid metabolism. In addition, co-exposure to other environmental pollutants, interindividual differences in genetic susceptibility, gender, and age may contribute to variability in the biological response to TEs and the resulting cardiovascular risk. Figure 2 illustrates the potential interplay between TE exposure, epigenetic mechanisms, and ASCVD-related pathways.

### 5.2. Study Strengths, Study Limitations, and Future Perspectives

Several strengths of the available evidence should be acknowledged. Human epidemiological studies provide evidence of associations between TE exposure, epigenetic alterations, and cardiovascular-related phenotypes, while animal and cell-based studies offer complementary insights into the molecular mechanisms potentially underlying these relationships. Some findings have been supported by validation in independent human cohorts, and experimental studies have identified specific epigenetic alterations associated with changes in pathways related to ASCVD. The convergence of epidemiological and experimental findings, therefore, supports the biological plausibility of a link between TE exposure, epigenetic dysregulation, and atherosclerosis, although the strength of the evidence varies across individual TEs and epigenetic mechanisms.

Despite these strengths, important limitations must be considered when interpreting the findings. Human studies are still predominantly cross-sectional, and relatively few longitudinal investigations have evaluated whether TE exposure precedes epigenetic alterations and whether these alterations may contribute to vascular dysfunction or cardiovascular outcomes. This limits the ability to establish temporal relationships and to distinguish potential causal pathways from exposure-related or disease-related epiphenomena. Establishing causal relationships between TE exposure, epigenetic alterations, and cardiovascular outcomes remains challenging. Epigenetic changes may represent mediators of exposure-related effects, adaptive cellular responses, or consequences of disease-related processes, and the available evidence does not always allow these possibilities to be clearly distinguished.

Moreover, substantial heterogeneity exists in the biomarkers investigated, analytical approaches, biological matrices, and methods used to characterize both TE exposure and epigenetic alterations. The use of different biological matrices, including blood, plasma or serum, urine, and tissue-specific samples, may capture distinct aspects and time windows of exposure and biological response, further limiting comparability across studies. In particular, epigenetic alterations detected in peripheral blood may not necessarily reflect those occurring in vascular tissues directly involved in atherosclerosis. Furthermore, differences in blood cell composition may influence DNA methylation measurements, while circulating non-coding RNA profiles may reflect contributions from multiple tissues. Future studies should therefore prioritize standardized sampling and analytical procedures and, where feasible, integrate peripheral biomarkers with tissue-specific or cell-specific analyses to determine the extent to which circulating epigenetic signatures reflect biologically relevant processes in the vasculature.

Evidence on combined or multiple-TE exposures is similarly limited, despite the fact that environmental exposure generally occurs as complex mixtures rather than isolated elements. In real-world scenarios, individuals are exposed to multiple pollutants, including particulate matter, pesticides, polycyclic aromatic hydrocarbons, and endocrine-disrupting chemicals, which may interact with TEs and exert additive, synergistic, or antagonistic effects. Such interactions may result in biological responses that cannot be fully predicted from studies investigating individual contaminants in isolation. Cumulative and combined environmental exposures may contribute to epigenetic alterations, including changes in DNA methylation and non-coding RNA expression, potentially influencing long-term susceptibility to atherosclerosis. However, the extent to which these combined exposures affect epigenetic regulation and contribute to ASCVD remains insufficiently characterized [72]. Finally, the latency, persistence, and reversibility of TE-associated epigenetic alterations, as well as their dependence on the timing and duration of exposure, remain poorly characterized. Collectively, these limitations constrain the interpretation, comparability, and translational relevance of the available findings.

Addressing these gaps will require well-designed longitudinal studies integrating repeated TE exposure assessment with epigenetic profiling and cardiovascular phenotyping. Greater attention should be given to early-life exposure, multiple-TE exposure, and cellular and tissue-specific responses, as these factors may substantially influence epigenetic effects. Repeated sampling may help distinguish transient molecular responses from persistent epigenetic changes potentially involved in longer-term vascular remodeling and disease susceptibility. Further studies should also investigate whether nutritional factors affecting one-carbon metabolism, including folate and vitamin B12 availability and SAM levels, modify the epigenetic effects of TE exposure. As these factors influence the availability of methyl groups for DNA methylation, they may contribute to interindividual variability in TE-associated epigenetic responses. To date, their role as potential modifiers of TE-induced DNA methylation changes, and the extent to which these interactions influence ASCVD risk, remain insufficiently characterized.

Integrative multi-omic approaches combining epigenetic mechanisms, transcriptomics, proteomics, and metabolomics may further clarify how different molecular layers interact and converge on atherosclerosis-related pathways, moving beyond isolated associations toward the identification of coherent molecular pathways linking exposure to functional vascular phenotypes. Further research should investigate the potential role of extracellular vesicles, including exosomes, in mediating the intercellular transfer of miRNAs following TE exposure. Characterizing EV-associated miRNA profiles and evaluating their functional effects on vascular cells may, in fact, help clarify whether this mechanism contributes to the molecular pathways linking TE exposure to atherosclerosis.

Candidate epigenetic signatures should also be replicated in independent populations with different exposure profiles and demographic and cardiovascular risk characteristics to assess their robustness. Prospective studies should evaluate whether these signatures can identify early vascular alterations or predict cardiovascular outcomes, while functional studies in relevant cellular and animal models will be essential to determine whether specific epigenetic alterations are mechanistically involved in vascular injury or primarily reflect exposure. Integrating exposure intensity, duration, timing, and co-exposure patterns with epigenetic and clinical data may help in further distinguishing transient adaptive responses, persistent molecular signatures of exposure, and intermediate biological events linking TE exposure to cardiovascular disease.

Clarifying these temporal and mechanistic relationships will be essential to determine whether epigenetic dysregulation represents a relevant link between environmental TE exposure and ASCVD and whether specific signatures may ultimately have utility for exposure assessment, early detection, and risk stratification. From a clinical perspective, epigenetic alterations may serve as complementary biomarkers to conventional cardiovascular risk factors, potentially providing additional information on the biological effects of TE exposure and individual susceptibility to ASCVD. More broadly, these findings highlight the importance of considering environmental TE exposure alongside traditional and non-traditional cardiovascular risk factors to better understand the multifactorial nature of ASCVD. Integrating these factors into a comprehensive framework may help guide future research and inform the development of more effective cardiovascular prevention strategies.

## Figures and Tables

**Table 1:** Summary of included studies on toxic element exposure, epigenetic mechanisms, and cardiovascular-related outcomes.

Author, Year	Study Type	Toxic Element(s) Investigated	Epigenetic Mechanism	Main Findings
**Domingo-Relloso et al., 2022 [60]**	Human cohort study in vivo study	As	DNA methylation (DMPs/DMRs)	20 DMPs associated with incident CVD and 13 with CVD mortality; 11 DMPs replicated in three independent cohorts. Several loci were related to metabolism. Cross-species analysis identified concordant methylation changes involving 20 genes (DMRs) and 10 genes (DMPs).
**Makhani et al., 2025 [61]**	In vivo; single-cell multi-omics	As	Chromatin accessibility	Chronic As exposure altered chromatin accessibility in plaque macrophages, with distinct responses in resident-like and foam cell-like macrophages.
**Lin et al., 2020 [16]**	Human cohort study	Pb, Cd	Global DNA methylation	Pb associated with altered global DNA methylation and increased CIMT; Cd associated with increased CIMT.
**Riffo-Campos et al., 2018 [62]**	Human longitudinal cohort study in silico analysis	As, Cd, Sb, W	DNA methylation (DMRs)	Cd and W associated with increased carotid and femoral intima-media thickness; 46% overlap between genes near metal-associated and atherosclerosis-related DMRs.
**Song et al., 2019 [63]**	In vitro; THP-1 macrophages	As	DNA methylation; DNMT1	As increased DNMT1 activity and ABCA1 promoter methylation, resulting in reduced ABCA1 expression and impaired cholesterol efflux; global hypomethylation was also observed.
**Poirier & Vlasova, 2002 [64]**	Ex vivo/in vitro; rat hepatic nuclear extracts	Cd, Zn	DNA methyltransferase activity	Cd and Zn inhibited DNA methyltransferase activity; Cd-mediated inhibition depended on methyl-group availability.
**Ngalame et al., 2013 [65]**	In vivo; ApoE−/− mice, prenatal exposure	As	DNA methylation	Prenatal As increased methylation within the transcribed region of Hsp70 in 10-week-old offspring without altering global DNA methylation.
**Svoboda et al., 2021 [66]**	In vivo; adult mice, perinatal exposure	Pb	DNA methylation	Perinatal Pb induced locus-specific DNA methylation changes, with marked sex-specific patterns.
**Wang et al., 2025 [14]**	In vivo; ApoE−/− mice Human case–control study	Cd	miRNAs	Cd altered miR-30d-5p and miR-504-3p, affecting NCEH1 and CD36, cholesterol homeostasis, and foam-cell formation. Human data further linked miR-30d-5p to Cd exposure and ischemic stroke.
**States et al., 2012 [67]**	In vivo; ApoE−/− mice, prenatal exposure	As	miRNA-related regulatory changes	Prenatal As altered hepatic gene-expression networks and miRNAs targeting Hsp70-related transcripts in adult offspring.
**Gao et al., 2016 [68]**	In vitro; HUVECs	As	miRNA	As reduced miR-425-5p and impaired endothelial proliferation, migration, and tube formation.
**González-Bravo et al., 2026 [69]**	Human biomonitoring/bioinformatic network	Pb	miRNAs; target-gene networks	Pb exposure associated with circulating miR-126 and miR-155; predicted targets involved in inflammation, angiogenesis, lipid metabolism, and atherosclerosis.
**Kong et al., 2012 [70]**	Human case–control study	As, Hg, Cd, Pb	miRNAs	Urinary As and Pb negatively associated with miR-21 and miR-221; miR-21 associated with microalbuminuria.
**Nguyen & Kim, 2023 [71]**	In silico	Cd, Pb, Hg	miRNAs, target genes, transcription factors	Combined Cd, Pb, and Hg exposure associated in silico with miRNA–target gene–transcription factor networks involving oxidative stress, inflammation, lipid metabolism, and IL-6 signalling.

Abbreviations: ABCA1, ATP-binding cassette transporter A1; ApoE−/−, apolipoprotein E knockout; As, arsenic; Cd, cadmium; CD36, cluster of differentiation 36; CIMT, carotid intima-media thickness; CVD, cardiovascular disease; DMPs, differentially methylated positions; DMRs, differentially methylated regions; DNMT1, DNA methyltransferase 1; Hg, mercury; Hsp70, heat shock protein 70; HUVEC, human umbilical vein endothelial cells; IL-6, interleukin-6; miRNA, microRNA; NCEH1, neutral cholesterol ester hydrolase 1; p38, p38 mitogen-activated protein kinase; Pb, lead; ROS, reactive oxygen species; Sb, antimony; THP-1, human monocytic cell line; VEGF, vascular endothelial growth factor; W, tungsten; Zn, zinc.

## Data Availability

No new data were created or analyzed in this study. Data sharing is not applicable to this article.

## Abbreviations

The following abbreviations are used in this manuscript:

As	Arsenic
ASCVD	Atherosclerotic cardiovascular disease
ABCA1	ATP-binding cassette transporter A1
Cd	Cadmium
CIMT	Carotid intima-media thickness
circRNA	Circular RNA
CVD	Cardiovascular disease
DMP	Differentially methylated position
DMR	Differentially methylated region
DNMT1	DNA methyltransferase 1
HBM	Human biomonitoring
Hg	Mercury
IHD	Ischemic heart disease
lncRNA	Long non-coding RNA
miRNA	microRNA
PAD	Peripheral artery disease
Pb	Lead
ROS	Reactive oxygen species
SAM	S-adenosylmethionine
TE	Toxic element
WHO	World Health Organization

## References

[1] Tsao C.W., Aday A.W., Almarzooq Z.I., Anderson C.A.M., Arora P., Avery C.L., Baker-Smith C.M., Beaton A.Z., Boehme A.K., Buxton A.E. (2023). Heart Disease and Stroke Statistics-2023 Update: A Report from the American Heart Association. Circulation.

[2] Yang A.M., Lo K., Zheng T.Z., Yang J.L., Bai Y.N., Feng Y.Q., Cheng N., Liu S.M. (2020). Environmental heavy metals and cardiovascular diseases: Status and future direction. Chronic Dis. Transl. Med..

[3] Palazzo M., Borghini A., Bustaffa E., Baldacci S., Gorini F., Minichilli F. (2026). Genetic Polymorphisms as Modifiers of Health Risks from Exposure to Toxic Elements: A Traditional Literature Review. Toxics.

[4] Lamas G.A., Bhatnagar A., Jones M.R., Mann K.K., Nasir K., Tellez-Plaza M., Ujueta F., Navas-Acien A., American Heart Association Council on Epidemiology and Prevention, Council on Cardiovascular and Stroke Nursing (2023). Contaminant Metals as Cardiovascular Risk Factors: A Scientific Statement From the American Heart Association. J. Am. Heart Assoc..

[5] Cheema S., Hussain S.I., Faheem M.S.B., Jalal A.A., Rifai M., Dar A., Burhan M., Shahid A., Ali M.S., Anwar A. (2025). Toxic Heavy Metal Exposure and Heart Health: A Systematic Review and Meta-Analysis of 324,331 Patients. BMC Cardiovasc. Disord..

[6] Liu X., Zhang D., Wu X., Tu J., Gong C., Li Y., Cui W., Chen J., Lu S. (2022). Urinary metals as influencing factors of coronary heart disease among a population in Guangzhou, China. Ecotoxicol. Environ. Saf..

[7] Baldacci S., Bustaffa E., Curzio O., Borghini A., Donzelli G., Cavigli C., Minichilli F. (2026). Environmental Toxic Metals and Metalloids Exposure and Cardiovascular Health: Current Evidence from Oxidative Stress and Inflammatory Biomarkers as Indicators of Subclinical Cardiovascular Effects. Antioxidants.

[8] Romanò A., Pagiatakis C., Gornati R., Bernardini G., Papait R. (2025). Epigenetics: A link between toxicants and diseases. iScience.

[9] Huang J., El-Kersh K., Mann K.K., James K.A., Cai L. (2022). Overview of the cardiovascular effects of environmental metals: New preclinical and clinical insights. Toxicol. Appl. Pharmacol..

[10] Zoghbi H.Y., Beaudet A.L. (2016). Epigenetics and Human Disease. Cold Spring Harb. Perspect. Biol..

[11] Arzua T., Jiang C., Yan Y., Bai X. (2021). The importance of non-coding RNAs in environmental stress-related developmental brain disorders: A systematic review of evidence associated with exposure to alcohol, anesthetic drugs, nicotine, and viral infections. Neurosci. Biobehav. Rev..

[12] Schmidt S. (2024). Marking Time: Epigenetic Aging May Partially Explain the Arsenic-Cardiovascular Disease Link. Environ. Health Perspect..

[13] Ryu H.W., Lee D.H., Won H.R., Kim K.H., Seong Y.J., Kwon S.H. (2015). Influence of toxicologically relevant metals on human epigenetic regulation. Toxicol. Res..

[14] Wang T., Lv Z., Fu X., Zhang Y., Zou D., Chen J., Zou Z., Liu J., Wang C., Xiong Y. (2025). Cadmium Exposure Promotes Atherosclerosis by Disrupting Cholesterol Homeostasis via miR-30d-5p Regulation. Environ. Sci. Technol..

[15] Khan F., Momtaz S., Abdollahi M. (2019). The relationship between mercury exposure and epigenetic alterations regarding human health, risk assessment and diagnostic strategies. J. Trace Elem. Med. Biol..

[16] Lin C.Y., Lee H.L., Hwang Y.T., Huang P.C., Wang C., Sung F.C., Wu C., Su T.C. (2020). Urinary heavy metals, DNA methylation, and subclinical atherosclerosis. Ecotoxicol. Environ. Saf..

[17] Iyer M., Anand U., Thiruvenkataswamy S., Babu H.W.S., Narayanasamy A., Prajapati V.K., Tiwari C.K., Gopalakrishnan A.V., Bontempi E., Sonne C. (2023). A review of chromium (Cr) epigenetic toxicity and health hazards. Sci. Total Environ..

[18] Sharma B.M., Komprdová K., Lörinczová K., Kuta J., Přibylová P., Scheringer M., Šebejová L., Piler P., Zvonař M., Klánová J. (2025). Human biomonitoring of essential and toxic trace elements (heavy metals and metalloids) in urine of children, teenagers, and young adults from a Central European Cohort in the Czech Republic. J. Expo. Sci. Environ. Epidemiol..

[19] Jomova K., Alomar S.Y., Nepovimova E., Kuca K., Valko M. (2025). Heavy metals: Toxicity and human health effects. Arch. Toxicol..

[20] Mustapha L.S., Obayami O.V., Obayami K.S. (2026). A comprehensive review on potential heavy metals in the environment: Persistence, bioaccumulation, ecotoxicology, and agricultural impacts. Ecol. Front..

[21] Pan Z., Gong T., Liang P. (2024). Heavy Metal Exposure and Cardiovascular Disease. Circ. Res..

[22] Agency for Toxic Substances and Disease Registry Substance Priority List. https://www.atsdr.cdc.gov/programs/substance-priority-list.html.

[23] World Health Organization Human Health Effects of Benzene, Arsenic, Cadmium, Nickel, Lead and Mercury: Report of an Expert Consultation. https://www.who.int/europe/publications/i/item/WHO-EURO-2023-8983-48755-72523.

[24] Esteban López M., Göen T., Mol H., Nübler S., Haji-Abbas-Zarrabi K., Koch H.M., Kasper-Sonnenberg M., Dvorakova D., Hajslova J., Antignac J.P. (2021). The European human biomonitoring platform—Design and implementation of a laboratory quality assurance/quality control (QA/QC) programme for selected priority chemicals. Int. J. Hyg. Environ. Health.

[25] Molina-Villalba I., Lacasaña M., Rodríguez-Barranco M., Hernández A.F., Gonzalez-Alzaga B., Aguilar-Garduño C., Gil F. (2015). Biomonitoring of arsenic, cadmium, lead, manganese and mercury in urine and hair of children living near mining and industrial areas. Chemosphere.

[26] Narayanan M. (2026). Heavy metal exposure among industrial workers mechanisms of toxicity biomonitoring strategies and public health risk assessment. Discov. Public Health.

[27] Sadee B.A., Galali Y., Zebari S.M.S. (2023). Toxicity, arsenic speciation and characteristics of hyphenated techniques used for arsenic determination in vegetables. A review. RSC Adv..

[28] IARC, International Agency for Research on Cancer (2012). Arsenic, Metals, Fibres, and Dusts. https://publications.iarc.who.int/Book-And-Report-Series/Iarc-Monographs-On-The-Identification-Of-Carcinogenic-Hazards-To-Humans/Arsenic-Metals-Fibres-And-Dusts-2012.

[29] WHO, World Health Organization (2022). Arsenic. https://www.who.int/news-room/fact-sheets/detail/arsenic.

[30] Yu Q., Wang W., Wu Z., Sun B., Zhang A. (2023). Association between exposure to arsenic and the risk of cardiovascular disease: Potential role of vascular endothelial injury. Environ. Toxicol. Pharmacol..

[31] Zhao J., Li A., Mei Y., Zhou Q., Li Y., Li K., Xu Q. (2021). The association of arsenic exposure with hypertension and blood pressure: A systematic review and dose-response meta-analysis. Environ. Pollut..

[32] Moon K., Guallar E., Navas-Acien A. (2012). Arsenic exposure and cardiovascular disease: An updated systematic review. Curr. Atheroscler. Rep..

[33] Mateen F.J., Grau-Perez M., Pollak J.S., Moon K.A., Howard B.V., Umans J.G., Best L.G., Francesconi K.A., Goessler W., Crainiceanu C. (2017). Chronic arsenic exposure and risk of carotid artery disease: The Strong Heart Study. Environ. Res..

[34] Nezu T., Hosomi N., Aoki S., Matsumoto M. (2016). Carotid Intima-Media Thickness for Atherosclerosis. J. Atheroscler. Thromb..

[35] Charkiewicz A.E., Omeljaniuk W.J., Nowak K., Garley M., Nikliński J. (2023). Cadmium Toxicity and Health Effects—A Brief Summary. Molecules.

[36] Rasin P., Ashwathi A.V., Basheer S.M., Haribabu J., Santibanez J.F., Garrote C.A., Arulraj A., Mangalaraja R.V. (2025). Exposure to Cadmium and Its Impacts on Human Health: A Short Review. J. Hazard. Mater. Adv..

[37] Wang M., Chen Z., Song W., Hong D., Huang L., Li Y. (2021). A review on Cadmium Exposure in the Population and Intervention Strategies Against Cadmium Toxicity. Bull. Environ. Contam. Toxicol..

[38] Cirovic A., Satarug S. (2024). Toxicity Tolerance in the Carcinogenesis of Environmental Cadmium. Int. J. Mol. Sci..

[39] Genchi G., Sinicropi M.S., Lauria G., Carocci A., Catalano A. (2020). The Effects of Cadmium Toxicity. Int. J. Environ. Res. Public Health.

[40] Verzelloni P., Urbano T., Wise L.A., Vinceti M., Filippini T. (2024). Cadmium exposure and cardiovascular disease risk: A systematic review and dose-response meta-analysis. Environ. Pollut..

[41] Tinkov A.A., Filippini T., Ajsuvakova O.P., Skalnaya M.G., Aaseth J., Bjørklund G., Gatiatulina E.R., Popova E.V., Nemereshina O.N., Huang P.T. (2018). Cadmium and atherosclerosis: A review of toxicological mechanisms and a meta-analysis of epidemiologic studies. Environ. Res..

[42] Raj K., Das A.P. (2023). Lead Pollution: Impact on Environment and Human Health and Approach for a Sustainable Solution. Environ. Chem. Ecotoxicol..

[43] Huang H., Guan H., Tian Z.-Q., Chen M.-M., Tian K.-K., Zhao F.-J., Wang P. (2024). Exposure Sources, Intake Pathways and Accumulation of Lead in Human Blood. Soil Secur..

[44] Angrand R.C., Collins G., Landrigan P.J., Thomas V.M. (2022). Relation of blood lead levels and lead in gasoline: An updated systematic review. Environ. Health.

[45] IARC, International Agency for Research on Cancer IARC Monographs on the Identification of Carcinogenic Hazards to Humans. https://monographs.iarc.who.int/agents-classified-by-the-iarc/.

[46] Navas-Acien A., Guallar E., Silbergeld E.K., Rothenberg S.J. (2007). Lead exposure and cardiovascular disease--a systematic review. Environ. Health Perspect..

[47] Park S.K., Wang X., Lee S., Hu H. (2024). Do we underestimate risk of cardiovascular mortality due to lead exposure?. Sci. Total Environ..

[48] Chowdhury R., Ramond A., O’Keeffe L.M., Shahzad S., Kunutsor S.K., Muka T., Gregson J., Willeit P., Warnakula S., Khan H. (2018). Environmental toxic metal contaminants and risk of cardiovascular disease: Systematic review and meta-analysis. BMJ.

[49] Guldbrand C., Barregard L., Sallsten G., Forsgard N., Lundh T., Borné Y., Fagerberg B., Engström G., Bergström G., Harari F. (2024). Low-level exposure to lead and atherosclerosis in the carotid arteries: Results from the Swedish population-based cohort SCAPIS. Environ. Res..

[50] Kim M.K., Zoh K.D. (2012). Fate and transport of mercury in environmental media and human exposure. J. Prev. Med. Public Health.

[51] Pavithra K.G., SundarRajan P., Kumar P.S., Rangasamy G. (2023). Mercury sources, contaminations, mercury cycle, detection and treatment techniques: A review. Chemosphere.

[52] Chiang G., Kidd K.A., Díaz-Jaramillo M., Espejo W., Bahamonde P., O’Driscoll N.J., Munkittrick K.R. (2021). Methylmercury biomagnification in coastal aquatic food webs from western Patagonia and western Antarctic Peninsula. Chemosphere.

[53] Minamata Convention of Mercury. https://minamataconvention.org/en/about.

[54] UN Environment Programme Minamata Convention COP-6 Agrees to End the Use of Dental Amalgam by 2034. https://www.unep.org/news-and-stories/press-release/minamata-convention-cop-6-agrees-end-use-dental-amalgam-2034.

[55] US EPA, United States Environmental Protection Agency Mercury in Dental Amalgam. https://www.epa.gov/mercury/mercury-dental-amalgam.

[56] Hu X.F., Lowe M., Chan H.M. (2021). Mercury exposure, cardiovascular disease, and mortality: A systematic review and dose-response meta-analysis. Environ. Res..

[57] Ozarde Y., Purandare D., Deshmukh S., Gadhave R. (2025). Heavy metals and cardiovascular health: Uncovering links and health challenges. J. Trace Elem. Med. Biol..

[58] Hocher B. (2026). Metal exposure in heart disease. Clin. Chim. Acta.

[59] Damiani I., Solberg E.H., Iyer M., Cheng P., Weldy C.S., Kim J.B. (2025). Environmental pollutants and atherosclerosis: Epigenetic mechanisms linking genetic risk and disease. Atherosclerosis.

[60] Domingo-Relloso A., Makhani K., Riffo-Campos A.L., Tellez-Plaza M., Klein K.O., Subedi P., Zhao J., Moon K.A., Bozack A.K., Haack K. (2022). Arsenic Exposure, Blood DNA Methylation, and Cardiovascular Disease. Circ. Res..

[61] Makhani K., Yang X., Dierick F., Subramaniam N., Gagnon N., Ebrahimian T., Lehoux S., Wu H., Ding J., Mann K.K. (2025). Single-Cell Multi-Omics Profiling of Immune Cells Isolated from Atherosclerotic Plaques in Male ApoE Knockout Mice Exposed to Arsenic. Environ. Health Perspect..

[62] Riffo-Campos A.L., Fuentes-Trillo A., Tang W.Y., Soriano Z., De Marco G., Rentero-Garrido P., Adam-Felici V., Lendinez-Tortajada V., Francesconi K., Goessler W. (2018). In silico epigenetics of metal exposure and subclinical atherosclerosis in middle aged men: Pilot results from the Aragon Workers Health Study. Philos. Trans. R. Soc. Lond. B Biol. Sci..

[63] Song Y., Zhou T., Zong Y., Gu B., Tan X., Yang L. (2019). Arsenic inhibited cholesterol efflux of THP-1 macrophages via ROS-mediated ABCA1 hypermethylation. Toxicology.

[64] Poirier L.A., Vlasova T.I. (2002). The prospective role of abnormal methyl metabolism in cadmium toxicity. Environ. Health Perspect..

[65] Ngalame N.N., Micciche A.F., Feil M.E., States J.C. (2013). Delayed temporal increase of hepatic Hsp70 in ApoE knockout mice after prenatal arsenic exposure. Toxicol. Sci..

[66] Svoboda L.K., Wang K., Jones T.R., Colacino J.A., Sartor M.A., Dolinoy D.C. (2021). Sex-Specific Alterations in Cardiac DNA Methylation in Adult Mice by Perinatal Lead Exposure. Int. J. Environ. Res. Public Health.

[67] States J.C., Singh A.V., Knudsen T.B., Rouchka E.C., Ngalame N.O., Arteel G.E., Piao Y., Ko M.S. (2012). Prenatal arsenic exposure alters gene expression in the adult liver to a proinflammatory state contributing to accelerated atherosclerosis. PLoS ONE.

[68] Gao Y., Yin Y., Xing X., Zhao Z., Lu Y., Sun Y., Zhuang Z., Wang M., Ji W., He Y. (2016). Arsenic-induced anti-angiogenesis via miR-425-5p-regulated CCM3. Toxicol. Lett..

[69] González-Bravo A., Rangel-Ramírez V.V., de la Trinidad-Chacón C.G., López-Ramírez M.L., Almendárez-Reyna C.I., Salazar-Rodríguez C.B., Carrizales-Yáñez L., Pérez-Maldonado I.N., Ochoa-Martínez Á.C. (2026). Circulating miR-126 and miR-155 are associated with environmental lead exposure: A translational approach via human biomonitoring and bioinformatic networks. Toxicol. Rep..

[70] Kong A.P., Xiao K., Choi K.C., Wang G., Chan M.H., Ho C.S., Chan I., Wong C.K., Chan J.C., Szeto C.C. (2012). Associations between microRNA (miR-21, 126, 155 and 221), albuminuria and heavy metals in Hong Kong Chinese adolescents. Clin. Chim. Acta.

[71] Nguyen H.D., Kim M.S. (2023). The Effects of a Mixture of Cadmium, Lead, and Mercury on Metabolic Syndrome and Its Components, as well as Cognitive Impairment: Genes, MicroRNAs, Transcription Factors, and Sponge Relationships. Biol. Trace Elem. Res..

[72] Gorini F., Palazzo M., Simonini L., Tonacci A., Rizza A., Wu H., Minichilli F., Borghini A. (2026). A Perfect Storm of Pollutants: Environmental Mixtures in the Pathogenesis of Atherosclerosis. Int. J. Mol. Sci..

